# Dynamically grading cerebral collateral circulation using 3D multi-inversion time arterial spin labeling in ischemic stroke: a comparison with digital subtraction angiography

**DOI:** 10.3389/fneur.2025.1553216

**Published:** 2025-08-29

**Authors:** Chuili Kong, Quanzhi Feng, Tiantian Yang, Shuwang Qiao, Xianchang Zhang, Josef Pfeuffer, Tong Han

**Affiliations:** ^1^Department of Radiology, Tianjin Huanhu Hospital, Tianjin, China; ^2^Department of Radiology, Central Hospital Affiliated to Shandong First Medical University, Jinan, China; ^3^Department of Radiology, Zhangqiu District People's Hospital, Jinan, China; ^4^MR Collaboration, Siemens Healthcare Ltd., Beijing, China; ^5^MR Application Development, Siemens Healthcare GmbH, Erlangen, Germany; ^6^Tianjin Key Laboratory of Cerebral Vascular and Neurodegenerative Diseases, Tianjin, China

**Keywords:** dynamic perfusion imaging, arterial spin labeling, collateral circulation, collateral grade, ischemic stroke

## Abstract

**Objective:**

This study aims to introduce a novel non-invasive imaging method, 3D multi-inversion time arterial spin labeling (3D mTI-ASL), for grading collateral circulation in patients with acute ischemic stroke (AIS) and to compare its effectiveness with digital subtraction angiography (DSA).

**Methods:**

We analyzed data from 28 patients with unilateral internal carotid artery or middle cerebral artery occlusion who underwent both DSA and 3D mTI-ASL imaging before endovascular treatment. A post-processing pipeline was established to grade collateral flow based on 16 perfusion-weighted images. The agreement between the collateral grades derived from ASL and DSA was evaluated.

**Results:**

Strong interobserver agreement was observed for both DSA (*κ* = 0.856) and mTI-ASL (*κ* = 0.849). A moderate level of consistency in collateral grading between 3D mTI-ASL and DSA was noted (*κ* = 0.568, *p* < 0.001). This was improved when patients were categorized into poor and good collateral groups within both grading systems (*k* = 0.781, *p* < 0.001). Notably, patients with good collaterals (Grades 3–4) had significantly lower admission National Institutes of Health Stroke Scale scores compared to patients with poor collaterals (*p* < 0.028 for DSA and *p* < 0.015 for mTI-ASL).

**Conclusion:**

The 3D mTI-ASL technique shows promise as a reliable, non-invasive method for assessing collateral circulation in AIS. By simulating DSA-derived grading, it may serve as a complementary tool in clinical decision-making for acute stroke management.

## 1 Introduction

Stroke is a leading cause of death worldwide and significantly contributes to years of life lost to disability. Acute ischemic stroke (AIS) accounts for approximately 65.3% of all stroke cases worldwide (1). Notably, large blood vessel occlusion (LVO), predominantly occurring in the anterior circulation, accounts for 29.3% of AIS cases, with an annual incidence of 24 cases per 100,000 individuals (2, 3). Collateral blood circulation plays a crucial role in supporting cerebral circulation among patients with ischemic cerebrovascular disease characterized by LVO (4). Growing evidence indicates that robust collateral status is associated with improved recanalization following mechanical thrombectomy, reduced risk of hemorrhagic transformation, and a smaller rate of infarct growth, as well as enhanced functional outcomes (5–8). Therefore, the rapid and reliable assessment of collateral status is of great significance in clinical practice for patients with AIS caused by LVO.

Digital subtraction angiography (DSA) is considered the gold standard for evaluating collateral circulation; however, its clinical use is limited due to being relatively costly, time-consuming, and invasive, which increases the associated risks (9). Other imaging techniques, such as computed tomography angiography, computed tomography perfusion, and dynamic susceptibility contrast-enhanced magnetic resonance perfusion, can also be used to assess collateral flow (10–12). For example, De Havenon et al. first used dynamic susceptibility contrast-enhanced magnetic resonance perfusion source data to evaluate collateral grade through time-dependent characteristics (12). Subsequently, Kim et al. (11) demonstrated that there was good consistency between this method and DSA in evaluating the collateral grade. These studies have supported the feasibility of perfusion imaging in the evaluation of collateral circulation. However, these methods involve the use of exogenous contrast agents and expose patients to ionizing radiation. Thus, a simple, non-invasive, and contrast-free imaging technique is required for the evaluation of collateral flow.

Arterial spin labeling (ASL) is a non-invasive, non-contrast imaging technique that effectively evaluates vascular reactivity and cerebrovascular reserve capacity, making it advantageous for assessing vascular diseases (13, 14). A notable development in ASL is the three-dimensional multi-inversion time ASL (3D mTI-ASL), which quantifies multiple hemodynamic parameters by fitting perfusion-weighted images (PWI) from various inversion times (15). Compared to pseudo-continuous ASL (pCASL) with a single post-labeling delay (PLD), which has been used to evaluate collateral flow (16–19), 3D mTI-ASL captures a more comprehensive representation of perfusion dynamics, particularly beneficial in patients with AIS and LVO, where prolonged arterial transit times can hinder assessment (20). Moreover, it has been demonstrated that 3D mTI-ASL can quantify collateral circulation in a manner comparable to DSA (21). Thus, the present study aims to develop a method for assessing collateral flow through multi-phase PWI from 3D mTI-ASL and to clinically validate this approach by comparing it with DSA in patients with AIS due to LVO.

## 2 Materials and methods

### 2.1 Ethics statement

The local institutional review board approved the study, and written informed consent was obtained from all participants.

### 2.2 Study participants

This study collected patient data from the “Research on Key Technologies and Evaluation of Early Imaging of Acute Ischemic Stroke” trial conducted at the hospital between December 2017 and February 2019. The trial enrolled 153 patients with AIS who underwent MR imaging (MRI) and DSA. Inclusion criteria: (1) patients had a diagnosis of AIS with internal carotid artery occlusion (ICA) and/or proximal middle cerebral artery occlusion (MCA) of the M1 segment; (2) patients received no clinical intervention before 3D mTI-ASL and DSA; (3) patients with available MRI, ASL, and DSA images. Exclusion criteria: (1) confirmed AIS patients with arterial dissection, aneurysms, Moyamoya disease, or arthritis; (2) patients with imaging abnormalities in the posterior circulation or bilateral anterior circulation; (3) patients with poor image quality due to motion artifacts, noise, or artifacts from foreign bodies.

### 2.3 Data collection

For this study, only previously collected but unexamined data were included. In addition to MRI and DSA data, the demographic data (gender, age, etc.), vascular risk factors (advancing age, hypertension, diabetes, smoking, and hyperlipidemia), cardiovascular history, and admission National Institute of Health Stroke Scale (NIHSS) score were collected from all participants.

### 2.4 MRI protocol

All patients underwent scanning on a 3 T MAGNETOM Skyra scanner (Siemens Healthcare, Erlangen, Germany) with a 20-channel head coil before receiving DSA imaging. Diffusion-weighted imaging was conducted with the following parameters: repetition time (TR) of 5,900 ms, echo time (TE) of 81 ms, field of view (FOV) of 240 × 240 mm^2^, number of excitations of 3, slice thickness of 4 mm with a 1 mm inter-slice gap, and a total of 24 slices. The 3D time-of-flight magnetic resonance angiography was performed using TR of 22 ms and TE of 3.8 ms, with a FOV of 230 × 230 mm^2^ and a slice thickness of 0.65 mm with a 0.2 mm gap.

The mTI-ASL imaging was performed using a prototype sequence with the following parameters: 3D gradient and spin-echo readout imaging (22) with FAIR-Q2TIPS labeling (23); TR of 4,600 ms; TE of 22 ms; a slice thickness of 4 mm; FOV of 192 × 192 mm^2^; a total of 20 slices; bolus duration of 700 ms; 16 time intervals ranging from 480 to 4,080 ms in increments of 240 ms; and total acquisition time of 5:09 min including an M0 scan (24).

### 2.5 DSA examination

DSA was performed using a biplane cerebral angiographic system (Axiom Artis VBl1D; Siemens Healthcare) and a high-pressure syringe (MEDRAD MARK V PROVIS). The internal and external carotid arteries and bilateral vertebral arteries were examined. Imaging through the arterial, capillary, and venous phases was performed to evaluate slow-flowing collateral vessels.

### 2.6 Post-processing pipeline

**Figure 1:**
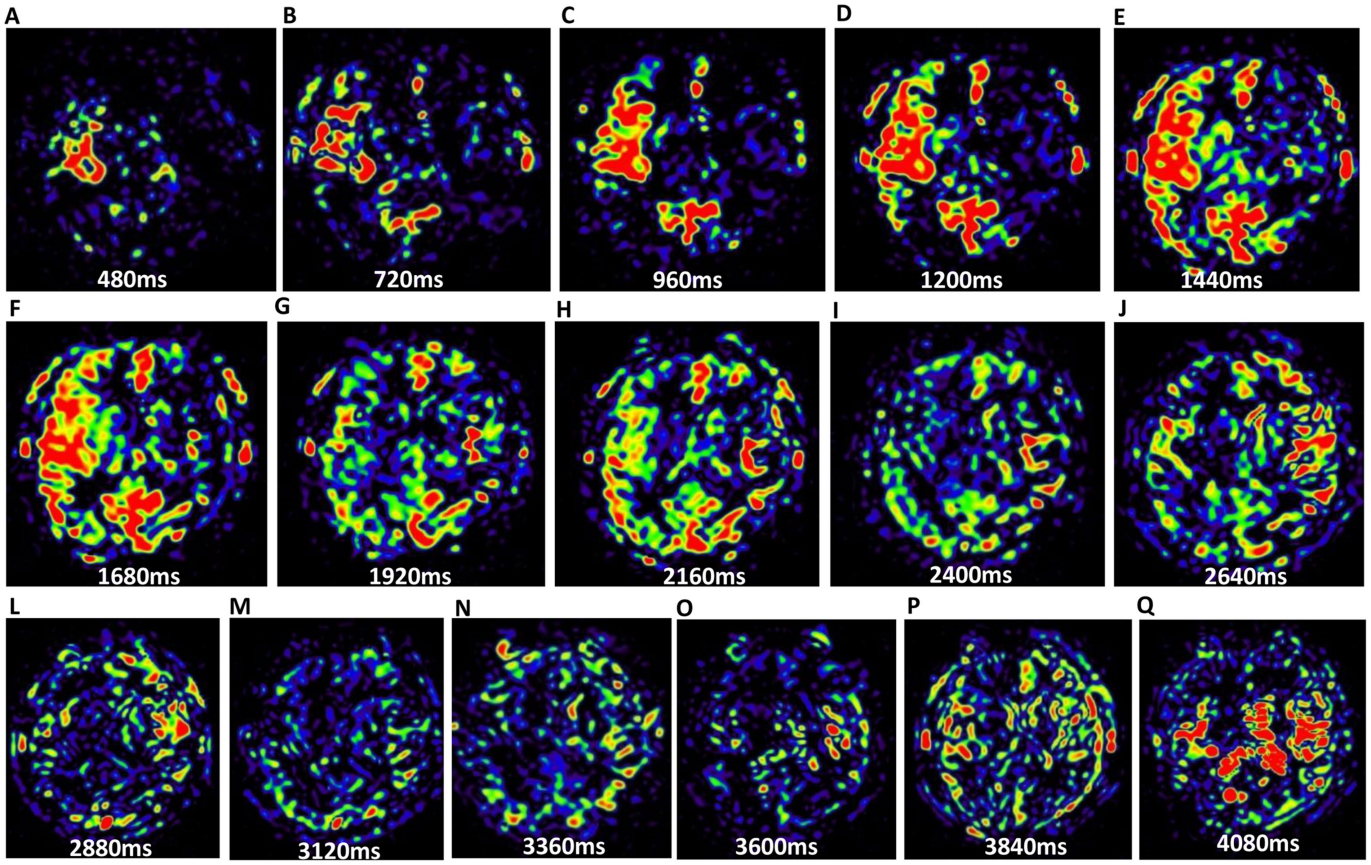
The 16 PWI maps of brain parenchyma from the same slice. **(A–Q)** Illustrate the progression from the first phase to the 16th phase. Variations in perfusion signal intensities across these periods indicate differences in cerebral blood flow states.

**Figure 2:**
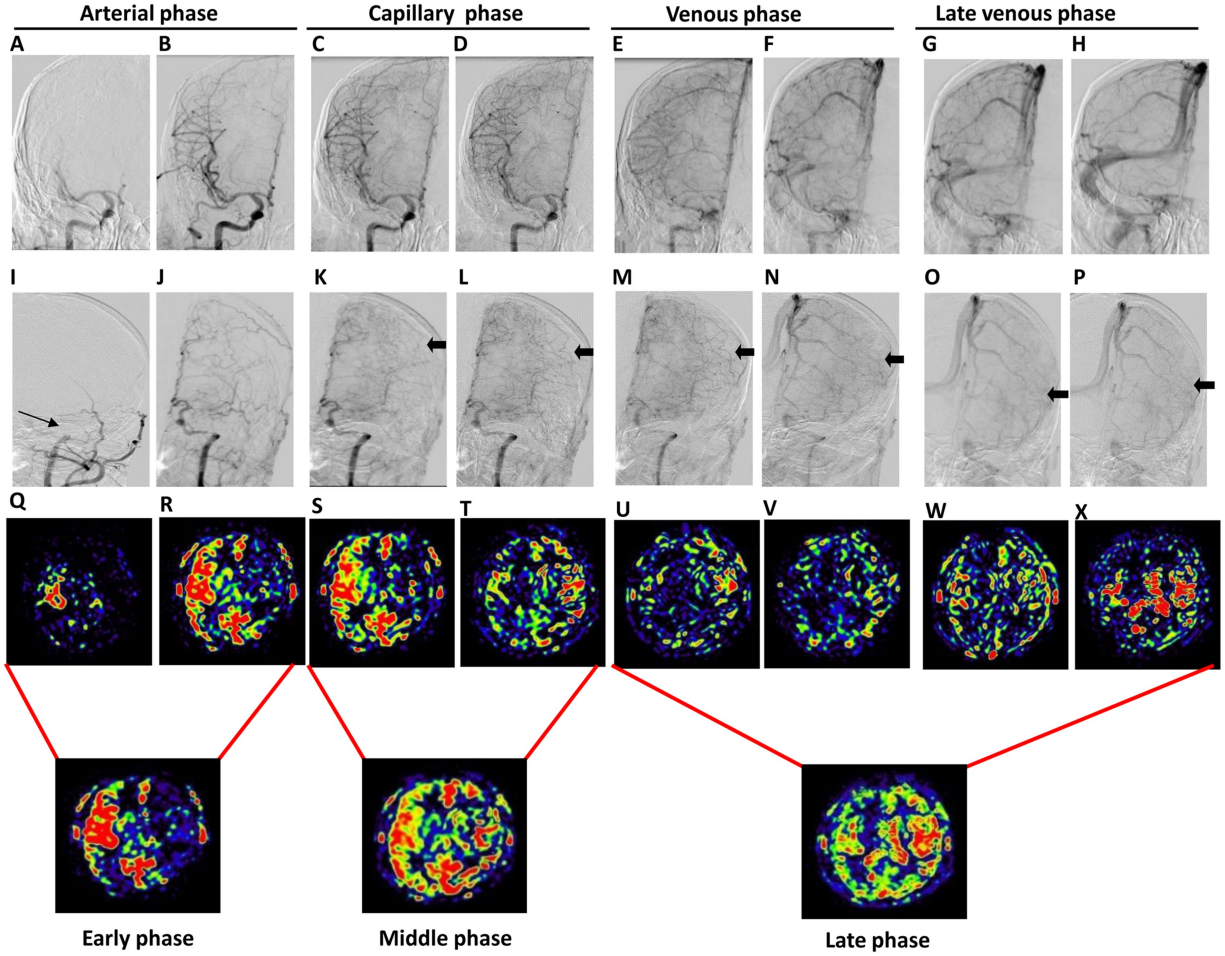
The occlusion of the left MCA in a 44-year-old male patient who experienced intermittent adverse right limb activity for over 20 days. The first two panels depict the anterior position DSA images of the lesion and affected sides, showing the arterial phase **(A,B,I,J)**, capillary phase **(C,D,K,L)**, venous phase **(E,F,M,N)**, and late venous phase **(G,H,O,P)**. These images illustrate a proximal occlusion of the left MCA (indicated by the thin arrow) accompanied by collateral compensation (indicated by the thick arrow). The third panel represents the PWI map, which includes the arterial phase **(Q,R)**, capillary phase **(S,T)**, venous phase **(U,V)**, and late venous phase **(W,X)**. Notably, cerebral blood flow on the affected side was delayed compared to that on the healthy side. The fourth panel illustrates the collateral flow map, which includes the early, middle, and late phases.

To evaluate collateral circulation, a neuroradiologist (CLK), who was blinded to patient information, performed post-processing of multiple PWI scans from 3D mTI-ASL. The post-processing pipeline comprised the following steps: (1) Image grouping: The 16 raw PWI images acquired during the 3D mTI-ASL sequence were organized into three distinct phases based on time-series analysis: early phase (images 1–5), middle phase (images 6–10), and late phase (images 11–16) (Figure 1). (2) Image overlay creation: Using the imaging workstation (syngo.via, Siemens Healthineers), the neuroradiologist employed the overlay function to superimpose all images within each phase to generate the corresponding overlay images: an early phase overlay image, a middle phase overlay image, and a late phase overlay image. (3) Correspondence with DSA phases: The early phase overlay image corresponded to the arterial phase in DSA, while the middle phase overlay image corresponded to the capillary phase, and the late phase overlay image corresponded to the venous/late venous phase in DSA (Figure 2). (4) Assessment of collateral state: Using the obtained three-phase images, referred to as the collateral flow maps based on mTI-ASL, collateral circulation was assessed according to the established grading criteria. Potential sources of variability in this assessment process included differences in arterial transit times across patients and any inconsistencies that may arise from variations in imaging parameters and post-processing techniques. Standardized imaging protocols were implemented to minimize such variability, and the neuroradiologist’s expertise in interpreting ASL-derived images also helped to ensure consistent grading.

### 2.7 Grading of the collateral circulation

The collateral circulation grading of DSA was based on the American Society of Interventional and Therapeutic Neuroradiology/Society of Interventional Radiology (ASITN/SIR) scale (25). There are five grades, ranging from Grade 0 to Grade 4. According to the ASITN/SIR scale for DSA, the grading of the collateral circulation for mTI-ASL was defined as follows: Grade 0 indicates no visible collaterals perfusion at the ischemic site; Grade 1 refers to slow collaterals (visible in the middle or late phase) reaching the periphery of the ischemic site, with persistent perfusion defects; Grade 2 denotes rapid collaterals (visible in the early-to-middle phase) extending to the ischemic site periphery, affecting only a portion of the ischemic territory and accompanied by persistent perfusion defects; Grade 3 describes slow but complete collateral perfusion to the ischemic bed; and Grade 4 indicates rapid and complete collateral perfusion throughout the entire ischemic territory. This grading system was applicable to both MCA and ICA occlusions.

Two neuroradiologists, QZF and TTY, each with over 8 years of experience in imaging diagnostics and blinded to the clinical information, independently performed the grading of the collateral circulation based on DSA and mTI-ASL images. Before the assessment, YSX, a clinician with 15 years of experience in interventional radiology, conducted a comprehensive one-week training for the neuroradiologists based on the ASITN/SIR Guidelines to ensure consistency in the grading process. Additionally, HT, the chief physician of the imaging department, provided a one-week training session following the ASL collateral circulation grade. Disagreements on the final results were resolved by discussion. Grades 0–2 were classified as poor collaterals, while Grades 3–4 were categorized as good collaterals (21).

### 2.8 Statistical analysis

Statistical analyses were performed using SPSS version 19.0 (IBM Corp., Armonk, NY, USA). The Kolmogorov–Smirnov and Shapiro–Wilk tests were used to assess data normality. Measurement data of normal distribution are expressed as means ± standard deviations. Qualitative data are presented as percentages and compared using Fisher’s exact test. The agreement between the two observers regarding DSA- and ASL-based collateral grades, as well as the consistency between 3D mTI-ASL and DSA final grades, was assessed using *kappa* statistics. The *к* coefficient was calculated. To assess the adequacy of our sample size and the statistical power of our analysis, we utilized PASS 2021 (v21.0.3) software for power analysis. *p* < 0.05 indicates statistical significance.

## 3 Results

### 3.1 Baseline clinical data

**Figure 3:**
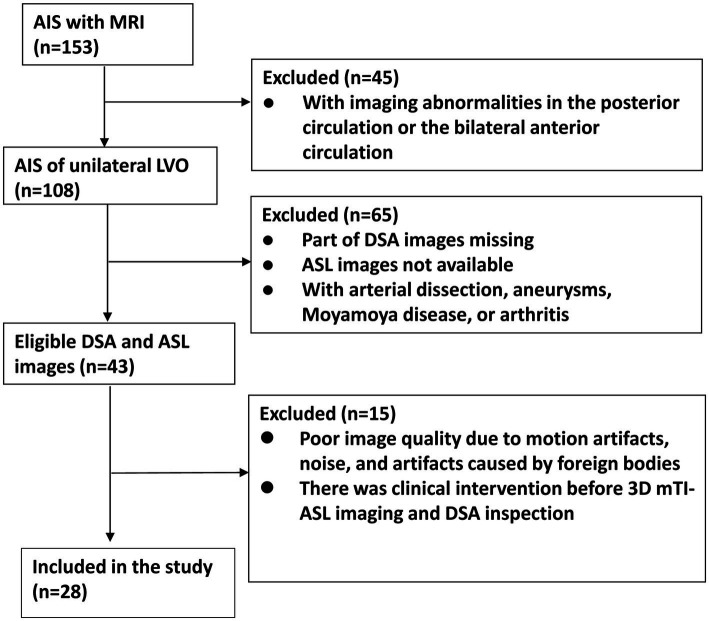
Flowchart of patient enrollment.

The flowchart of participant enrollment is presented in Figure 3. After screening using the inclusion and exclusion criteria, 28 patients were included in this study. The vast majority of patients were excluded due to the absence of DSA or ASL images. The results of the power analysis indicated that the sample size of 28 patients achieved a power of 96.7%, which is higher than the commonly accepted threshold of 90%. Thus, our sample size was sufficient for detecting statistical significance. The clinical data of the 28 patients are listed in Table 1. Among these patients, 16 had ICA occlusions, while 12 presented with MCA occlusions. There were 22 males and 6 females with a mean age of 58 ± 11 years. The primary vascular risk factors included abnormal lipid metabolism (60.7%), hypertension (71.4%), and the admission NIHSS score (4.36 ± 2.87). Additionally, we retrospectively categorized the stroke etiology in our patient cohort. Of the 28 patients evaluated, 17 (60.7%) were likely due to atherosclerosis, 7 (25.0%) were possibly cardioembolic, and 4 (14.3%) had undetermined origins, while no cases were classified under other causes. Notably, the diagnosis of ‘cardioembolic’ and ‘undetermined’ etiology often arose from incomplete evaluations due to patient constraints, such as financial limitations or a misunderstanding of the investigative process.

**Table 1:** Baseline clinical data of all patients.

Variables		Total (*n* = 28)
Age (years)		58 ± 11
Gender (male)		22 (78.6%)
Diabetes		7 (25.0%)
Hypertension		20 (71.4%)
Coronary heart disease		4 (14.3%)
Dyslipidemia		17 (60.7%)
Smoking (yes)		10 (35.7%)
Drinking (yes)		8 (28.6%)
NIHSS score at admission		4.36 ± 2.87
ICA occlusion		16 (57.1%)
MCA occlusion		12 (42.9%)
Stroke etiology	Atherosclerosis	17 cases (60.7%)
	Cardioembolic	7 cases (25.0%)
	Other causes	0 cases (0%)
	Undetermined etiology	4 cases (14.3%)

ICA, internal carotid artery; MCA, middle cerebral artery; NIHSS, National Institute of Health Stroke Scale.

### 3.2 Collateral circulation grading results

The collateral circulation grading results based on DSA images revealed that the numbers of patients with Grades 0 to 4 were 0, 6, 7, 8, and 7, respectively. The *к* coefficient for the interobserver agreement was 0.856. There were 13 patients with poor collaterals (Grades 0–2) and 15 patients with good collaterals (Grades 3–4) (Table 2). There were no statistically significant differences in gender, age, and vascular risk factors between poor- and good-collateral groups. The admission NIHSS score of the good collateral group was significantly lower than that of the poor collateral group (*p* = 0.028) (Table 2).

**Table 2:** Comparison of patients with different collateral grades based on DSA images.

Variables		Collateral grading based on DSA images		*p*
		Good collaterals (Grades 3–4) (*n* = 15)	Poor collaterals (Grades 0–2) (*n* = 13)	
Age (years)		58 ± 9	57 ± 14	0.827
Gender (male)		12 (80.0%)	10 (76.9%)	1.000
Diabetes		4 (26.7%)	3 (23.1%)	0.588
Hypertension		11 (73.3%)	9 (69.2%)	1.000
Coronary heart disease		3 (20.0%)	1 (7.69%)	0.600
Dyslipidemia		9 (60.0%)	8 (61.5%)	1.000
Smoker		6 (40.0%)	4 (30.8%)	0.705
Drinker		5 (33.3%)	3 (23.1%)	0.686
NIHSS score at admission		3.27 ± 2.58	5.61 ± 2.75	0.028
Stroke etiology	Atherosclerosis	11 (73.3%)	6 (46.2%)	0.230
	Cardioembolic	2 (13.3%)	5 (38.5%)	
	Other causes	0 (0%)	0 (0%)	
	Undetermined	2 (13.3%)	2 (15.4%)	

DSA, digital subtraction angiography; NIHSS, National Institute of Health Stroke Scale.

The collateral circulation was also graded based on the mTI-ASL images. The results indicated that there were 0, 7, 3, 11, and 7 patients classified as Grades 0, 1, 2, 3, and 4, respectively. The *к* coefficient for the interobserver agreement was 0.849. A total of 10 patients were graded with poor circulation (Grades 0–2), and 18 patients with good circulation (Grades 3–4). The admission NIHSS score of the good-collateral group was significantly lower than that of the poor-collateral group (*p* = 0.015) (Table 3).

**Table 3:** Comparison of patients with different collateral grades based on 3D mTI-ASL images.

Variables		Collateral grading based on 3D mTI-ASL images		*p*
		Good collaterals (Grades 3–4) (*n* = 18)	Poor collaterals (Grades 0–2) (*n* = 10)	
Age (years)		58 ± 9	57 ± 16	0.87
Gender (male)		14 (77.8%)	7 (70.0%)	0.67
Diabetes		5 (27.8%)	2 (20.0%)	1.00
Hypertension		14 (77.8%)	6 (60.0%)	0.40
Coronary heart disease		3 (16.7%)	1 (10.0%)	1.00
Dyslipidemia		11 (61.1%)	6 (60.0%)	1.00
Smoker		6 (33.3%)	4 (40.0%)	1.00
Drinker		5 (27.8%)	3 (30.0%)	1.00
NIHSS score at admission		3.44 ± 2.83	6.00 ± 2.21	0.015
Stroke etiology	Atherosclerosis	12 (66.7%)	5 (50%)	0.562
	Cardioembolic	3 (16.7%)	4 (40%)	
	Other causes	0 (0%)	0 (0%)	
	Undetermined	3 (16.7%)	1 (10%)	

3D mTI-ASL, three-dimensional multi-inversion time arterial spin labeling; NIHSS, National Institute of Health Stroke Scale.

To further explore the relationship between stroke etiology and collateral circulation, we conducted a comparative analysis between collateral grading and etiology under both the DSA and ASL classifications. As presented in Tables 2, 3, patients with atherosclerotic strokes generally exhibited better collateral circulation, whereas those with cardioembolic strokes showed poorer collateral flow. However, there was no significant difference (*p* > 0.05).

### 3.3 Consistency between two sets of collateral circulation grades

**Figure 4:**
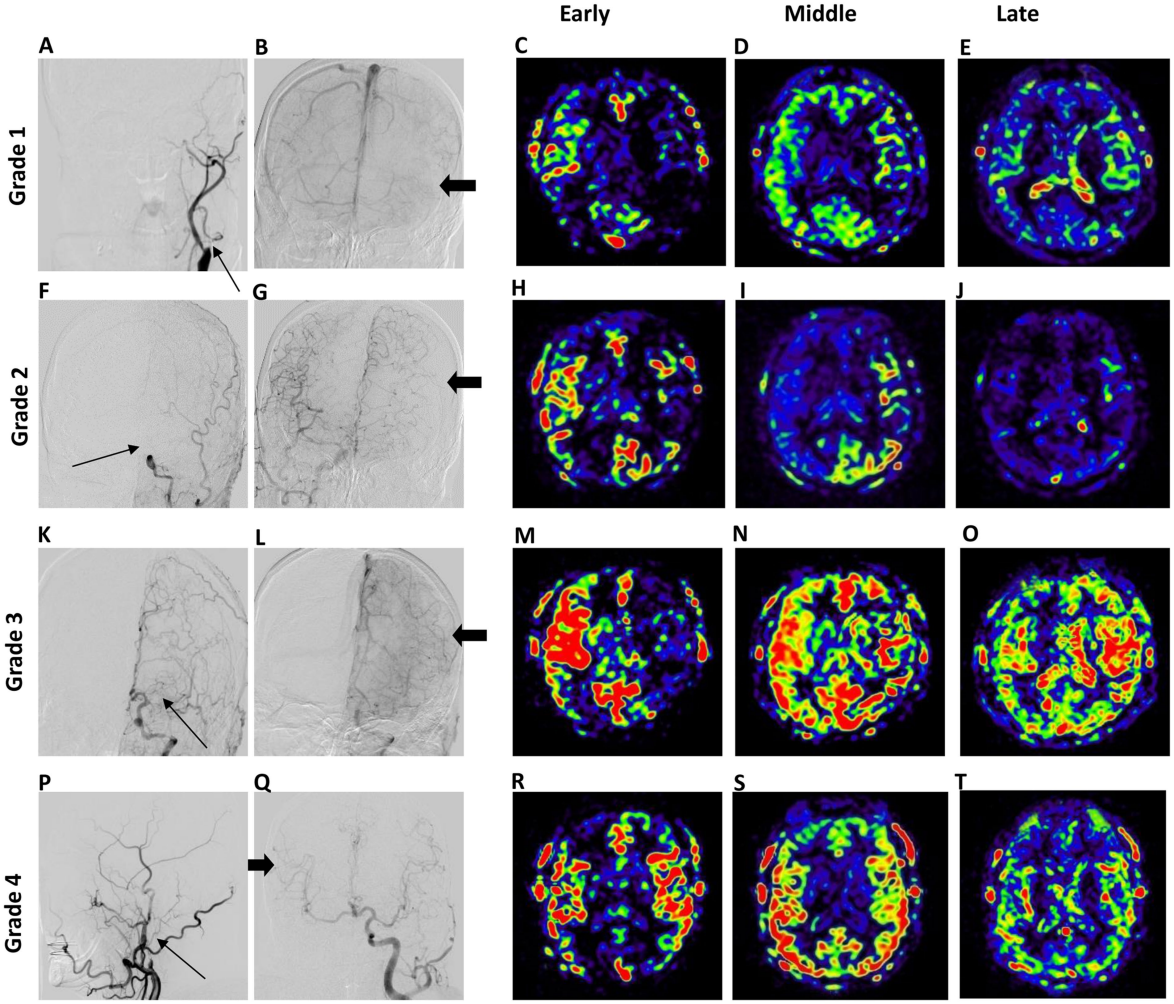
Typical cases of DSA-based (left panel) and collateral flow map-based (right panel) collateral grades. In Grade 1, the anterior DSA reveals left ICA occlusion (thin arrow) and inadequate collateral compensation during the venous phase of right ICA angiography **(A,B)**. The collateral flow map indicates low collateral compensation with a perfusion defect in the left middle cerebral artery (MCA) territory **(C,E)**. In Grade 2, the anterior DSA shows left ICA occlusion (arrow) alongside collateral compensation during the arterial phase of right ICA angiography (thick arrow) **(F,G)**. The collateral flow map demonstrates rapid collateral compensation, although a perfusion defect persists in the left MCA territory **(H–J)**. Grade 3 displays anterior DSA indicating left MCA occlusion with collateral flow from the anterior cerebral artery (thick arrow) during the venous phase **(K,L)**. The collateral flow map shows slow but complete compensatory perfusion in the left MCA territory **(M–O)**. In Grade 4, lateral DSA of the right carotid artery reveals right ICA occlusion (arrow) with collateral flow from left to right through the Circle of Willis during the arterial phase **(P,Q)**. The collateral flow map shows both rapid and complete collateral compensation in the right MCA territory **(R–T)**.

The grades of collateral circulation, as assessed by DSA and 3D mTI-ASL images, were compared. A total of 19 patients were categorized similarly by both collateral grading systems. Among these, five patients were rated as Grade 1 by both systems, three as Grade 2, seven as Grade 3, and four as Grade 4 (Table 4). There was a moderate consistency of agreement between DSA and 3D mTI-ASL (*к* = 0.568, *p* < 0.001) (Table 4). Typical cases of collateral circulation grade based on DSA and 3D mTI-ASL images are shown in Figure 4. The 3D mTI-ASL images accurately graded collateral circulation by reflecting dynamic perfusion velocities and the status of LVO, simulating DSA.

**Table 4:** The grading of collateral circulation by DSA and 3D mTI-ASL images.

Grade		ASL				Total
		Grade 1	Grade 2	Grade 3	Grade 4	
DSA	Grade 1	5	0	1	0	6
	Grade 2	2	3	0	2	7
	Grade 3	0	0	7	1	8
	Grade 4	0	0	3	4	7
Total		7	3	11	7	28
*к* coefficient		0.568				
*p*		<0.001				

3D mTI-ASL, three-dimensional multi-inversion time arterial spin labeling; DSA, digital subtraction angiography.

**Figure 5:**
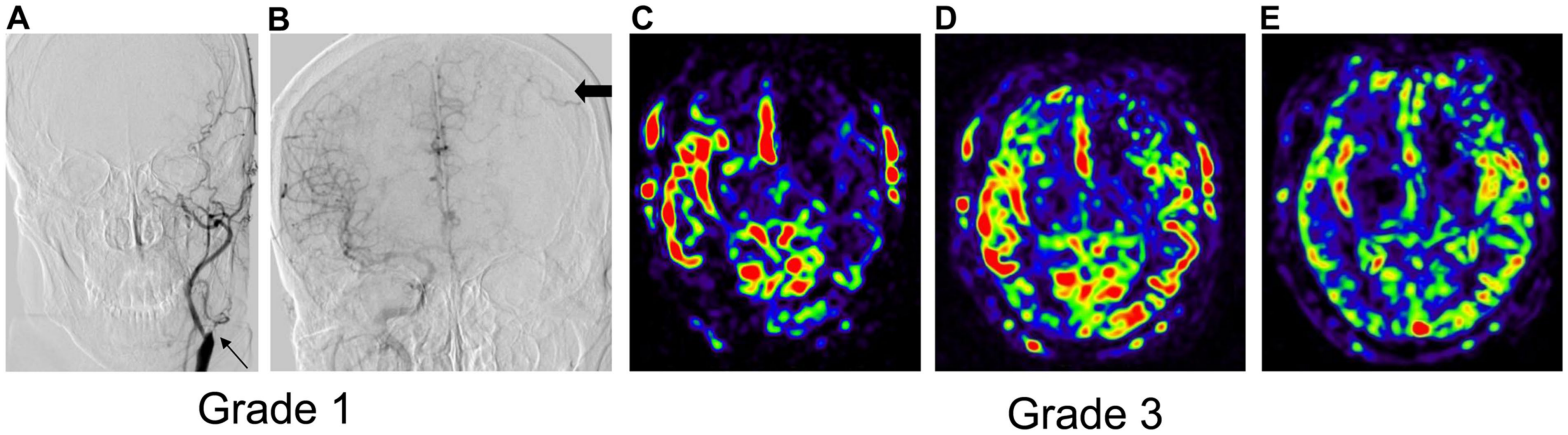
The images of a typical case with inconsistent collateral grading by DSA and 3D mTI-ASL. **(A)** The anterior DSA of left ICA. The thin arrow represents left ICA occlusion. **(B)** The anterior DSA of right ICA. The thick arrows indicate inadequate collateral compensation during the venous phase. **(C)** Significant hypoperfusion in the left MCA territory. **(D)** Partial hypoperfusion of the left frontal lobe in the left MCA territory. **(E)** Complete compensatory perfusion in the left MCA territory.

In addition, when the patients were divided into poor- and good-collateral groups, the consistency of collateral grading between DSA and 3D mTI-ASL was good (*к* = 0.781, *p* < 0.001) (Table 5). The collateral grading results for 25 patients were consistent, and 3 patients were inconsistent between the two methods. The images of a typical case with inconsistent collateral grading by DSA and 3D mTI-ASL are presented in Figure 5. In this case, the anterior DSA images showed left ICA occlusion and inadequate collateral compensation during the venous phase of right ICA angiography, indicating Grade 1 collateral status (Figures 5A,B). However, the corresponding collateral flow map derived from 3D mTI-ASL revealed slow but complete compensatory perfusion in the left MCA territory, suggesting Grade 3 collateral status (Figures 5C–E). In both methods, 15 patients were graded as having good collaterals, and 10 patients were graded as having poor collaterals (Table 5).

**Table 5:** Agreement between two sets of collateral circulation grades.

DSA	ASL		Total
	Good collaterals (Grades 3–4)	Poor collaterals (Grades 0–2)	
Good collaterals (Grades 3–4)	15	0	15
Poor collaterals (Grades 0–2)	3	10	13
Total	18	10	28
*к* coefficient	0.781		
*p*	<0.001		

3D mTI-ASL, three-dimensional multi-inversion time arterial spin labeling; DSA, digital subtraction angiography.

## 4 Discussion

Our study preliminarily demonstrated that the collateral status assessed by DSA and 3D mTI-ASL showed high consistency. The ASL imaging technique spatially defines the magnetic label that flows from the proximal arteries and accumulates in the distal microvasculature during the PLD and TI, similar to the transit of contrast observed during DSA (26). The current study obtained multiple PWI images with the 3D mTI-ASL by applying the marked technology of 16 inversion times. This technique demonstrates enhanced sensitivity to both temporal and perfusion information. These PWI images accurately depict the progression of labeled blood flowing from the supplying artery to the capillary network and ultimately returning to the venous sinus. Early PWI images from patients with internal carotid or middle cerebral artery occlusion showed inadequate anterior blood flow, which takes a shorter route to the target territory. However, mid to late-stage PWI images exhibited compensation through lateral blood flow, which travels longer and more circuitous paths (19, 27). The primary advantage of this study lies in its application of dynamic and macroscopic hemodynamic information obtained from ASL to evaluate collateral flow. The concept of collateral grading based on PWI images is fundamentally similar to that of DSA. The PWI images were structured into three post-processing stages. First, it was consistent with the arterial, capillary, and venous stages of DSA, thereby enhancing the clarity of the stage boundaries. Second, it was designed to meet clinical needs, facilitating more convenient evaluations. Third, the signal-to-noise ratio and spatial resolution are significantly improved, although they compromise time resolution. The results indicate that this straightforward and user-friendly method exhibits strong interobserver agreement and is consistent with DSA in assessing collateral status. Understanding collateral circulation status is crucial for clinicians when treating patients undergoing mechanical thrombectomy (28). Therefore, the proposed technique is non-invasive, user-friendly, and demonstrates considerable potential for evaluating collateral circulation in assessing perfusion improvement in patients with AIS due to LVO.

Collateral circulation is crucial in maintaining neural function during cerebral ischemia (4). Numerous clinical studies (29, 30) involving patients with comparable degrees of stenosis or occlusion have reported a spectrum of clinical manifestations, ranging from the absence of symptoms to transient ischemic attacks, even with some patients experiencing extensive cerebral infarctions. The variability in these clinical manifestations primarily depends on establishing adequate collateral circulation, which is essential for maintaining cerebral blood flow in areas at risk of ischemia (31, 32). This study revealed that the NIHSS score in patients with good collateral circulation was significantly lower than that of those with poor collateral circulation. Furthermore, no significant differences in age, gender, or risk factors were found between patients with good and poor collateral flow. Consequently, the influence of physiological variability on cerebral perfusion can be excluded. Furthermore, collateral circulation also plays a pivotal role in the prognosis of endovascular treatment. For example, Xu et al. have demonstrated that better collateral status is associated with improved clinical outcomes and higher rates of successful reperfusion in patients with AIS caused by LVO who underwent mechanical thrombectomy (33). A systematic review further supports this assertion, indicating that robust collateral vessels are linked to decreased infarct growth, greater success in recanalization efforts, lower rates of hemorrhagic transformation following endovascular treatment, and an extended therapeutic time window for revascularization (5). Hence, for patients undergoing mechanical thrombectomy, a favorable collateral status indicates a better prognosis.

In our study, the proposed method demonstrated a high level of consistency with DSA; however, the DSA and mTI-ASL grading systems generated inconsistent collateral statuses in nine patients. Further analysis of these nine cases revealed that 6 belonged to the same collateral status group and three belonged to different collateral status groups. The inconsistency may be attributed to the relatively lower temporal resolution associated with the PWI. Nevertheless, when both methods were used to assess collateral circulation status, a strong consistency was found between them. In the remaining three cases with conflicting collateral statuses (either poor or good) between mTI-ASL and DSA grading systems, the ASL results indicated a higher grading than the DSA results. The primary reason for this result may be that cerebral perfusion is affected by factors such as vascular dilatation, oxygen intake, blood pressure, and the patient’s ability to self-regulate. Thus, perfusion imaging may only capture the overall compensatory effects (34, 35). Another reason may be that subjective evaluation could impact the assessment of the combined effects of both anterior and posterior circulation and the compensation determined by DSA (25). Additionally, consistent with prior research (36), our analysis of stroke etiology and its relationship with collateral circulation indicated a trend wherein atherosclerotic strokes were associated with improved collateral circulation, while cardioembolic strokes tended to correlate with poorer collateral flow. However, due to the limited sample size, no significant differences were observed. In future studies, we intend to conduct prospective research with larger sample sizes to investigate these relationships more closely and discern their clinical implications.

Many studies have shown that ASL can accurately identify ischemic hypoperfusion areas and assess collateral circulation (13, 20). Zaharchuk et al. first revealed that pCASL with a single PLD could noninvasively predict the presence and intensity of collateral flow in patients with moyamoya disease (16). This technique has been further validated in patients with AIS and cerebrovascular stenosis, although varying machines and PLD were used (16–19). However, it often relies on a single PLD to assess hemodynamic parameters, which can limit the ability to capture dynamic changes in cerebral blood flow, particularly in cases of prolonged arterial arrival times, which are common in patients with LVO (20, 37). Another study demonstrated that subtraction images obtained from two delays of 3D pCASL (1.5 s and 2.5 s PLD) could quantitatively distinguish the role of collateral perfusion (27). However, compared to the ASITN/SIR of DSA, which dynamically assesses collateral grading from both temporal and spatial perspectives (25), the aforementioned methods do not evaluate collateral circulation in terms of dynamic perfusion. In contrast, the 3D mTI-ASL technique collects multiple PWI images at various inversion times, allowing for the simultaneous quantification of multiple hemodynamic parameters (15). This capability enhances the evaluation of collateral circulation by offering insights into both the arterial and venous phases of blood flow. Another promising ASL technique, vessel-selective or vessel-encoded ASL, labels the vessels of interest to evaluate antegrade and collateral flow (38, 39). Nonetheless, in both approaches, imperfect labeling efficiency must be considered when quantifying cerebral blood flow or mixed perfusion fractions (40). The cerebral collateral circulation involves not only the first-level circle of Willis, which provides rapid blood flow compensation, but also second-level sources such as the ophthalmic artery and leptomeningeal collateral branches, as well as third-level compensatory neovascularization, which plays a critical role in maintaining cerebral blood volume. Imperfect labeling may lead to an underestimation of collateral flow grades. Our findings indicate that 3D mTI-ASL may provide a more comprehensive assessment of collateral status compared to existing methods, as it mirrors the dynamic changes observed in DSA while eliminating the need for contrast agents. Due to the retrospective nature of the current study, we were unable to incorporate other ASL imaging techniques for direct comparison. However, the ability to generate collateral flow maps using 3D mTI-ASL establishes a promising foundation for future multi-center studies aimed at validating its clinical utility and comparison against other ASL variants. As the field of perfusion imaging continues to evolve, 3D mTI-ASL has the potential to emerge as a key tool for evaluating cerebral perfusion dynamics in various vascular diseases.

In addressing the limitations of DSA, it is crucial to consider the potential role of 3D mTI-ASL in clinical practice. The non-invasive, contrast-free characteristics of 3D mTI-ASL present a significant advancement, particularly for patients who may be at higher risk for complications from DSA. For instance, patients with kidney insufficiency or allergy to contrast agents may benefit substantially from this alternative assessment tool. Moreover, the rapid and reliable evaluation of collateral circulation using 3D mTI-ASL can greatly enhance clinical decision-making in AIS scenarios. The clinical treatment window for thrombectomy ranges from 6 to 24 h, as evidenced by the DAWN (Triage of Wake-up and Late Presenting Strokes Undergoing Neurointervention With Trevo) and DEFUSE 3 (Endovascular Therapy Following Imaging Evaluation for Ischemic Stroke 3) studies (41, 42). Both of these trials utilized the Rapid Processing of Perfusion and Diffusion automated software platform to determine the imaging eligibility of all patients. However, these techniques have limitations, as it is not feasible to ensure uniform use of the same software platform across all stroke centers. Therefore, a simple and timely imaging method for patient selection is needed, particularly for late-window thrombectomy. By providing timely and accurate diagnostics, 3D mTI-ASL can identify good collateral flow, making it an appropriate option for patient selection for late-window thrombectomy. Ultimately, while 3D mTI-ASL may not entirely replace DSA, it offers a valuable, complementary approach that can improve patient management pathways, particularly in high-stakes clinical settings, and guidance for late window thrombectomy.

This study has several limitations. First, the relatively small sample size of 28 patients may limit the generalizability of our findings, despite achieving statistical significance; larger cohort studies are necessary to validate these results. For example, future research could aim to include at least 200 patients by collaborating with multiple hospitals or research institutions to ensure a more diverse patient population. Second, the study did not consider cerebrovascular occlusions other than ICA and MCA occlusions, nor did it account for anatomical variations that could influence collateral circulation. Future studies should expand inclusion criteria to encompass a broader spectrum of cerebrovascular conditions, such as vertebrobasilar occlusions and other rare vascular anomalies. Third, cases with a collateral grade of 0 were excluded due to their low probability of occurrence, which may introduce selection bias. Future research should include these cases to provide a more comprehensive understanding of collateral circulation. Fourth, potential confounders, such as variations in patient demographics, comorbidities, and timing of imaging relative to stroke onset, could have influenced our assessments of collateral circulation. Future multicenter studies should systematically collect and analyze these demographic data to control for these variables in the statistical analysis. Fifth, our study used a qualitative assessment method for collateral grading, which limits our ability to calculate diagnostic sensitivity and specificity for the 3D mTI-ASL approach compared to DSA. Future studies should consider employing quantitative methodologies to evaluate collateral circulation and derive robust performance metrics, which would enhance the clinical applicability of the 3D mTI-ASL technique. Lastly, the retrospective nature of the study introduces inherent biases in patient selection and data interpretation. Therefore, a prospective design utilizing standardized imaging protocols across participating centers can help reduce variability. For instance, all centers could agree on specific ASL imaging sequences and timing protocols, as well as utilize a unified collateral grading scale, such as the ASITN/SIR classification.

In conclusion, the collateral flow map generated by 3D mTI-ASL, which simulates DSA, demonstrates high reliability in evaluating the grade of collateral circulation. This suggests that the proposed method may be a promising tool for directly providing valuable collateral flow information for AIS caused by LVO.

## Data Availability

The raw data supporting the conclusions of this article will be made available by the authors without undue reservation.

## References

[1] G. B. D. Stroke Risk Factor Collaborators. Global, regional, and national burden of stroke and its risk factors, 1990-2021: a systematic analysis for the global burden of disease study 2021. Lancet Neurol. (2024) 23:973–1003. doi: 10.1016/S1474-4422(24)00369-7, PMID: 39304265 PMC12254192

[2] Lakomkin N Dhamoon M Carroll K Singh IP Tuhrim S Lee J. Prevalence of large vessel occlusion in patients presenting with acute ischemic stroke: a 10-year systematic review of the literature. J Neurointerv Surg. (2019) 11:241–5. doi: 10.1136/neurintsurg-2018-014239, PMID: 30415226

[3] Rai AT Seldon AE Boo S Link PS Domico JR Tarabishy AR. A population-based incidence of acute large vessel occlusions and thrombectomy eligible patients indicates significant potential for growth of endovascular stroke therapy in the USA. J Neurointerv Surg. (2017) 9:722–6. doi: 10.1136/neurintsurg-2016-012515, PMID: 27422968 PMC5583675

[4] Liebeskind DS. Collateral circulation. Stroke. (2003) 34:2279–84. doi: 10.1161/01.STR.0000086465.41263.06, PMID: 12881609

[5] Lee JS Bang OY. Collateral status and outcomes after thrombectomy. Transl Stroke Res. (2023) 14:22–37. doi: 10.1007/s12975-022-01046-z, PMID: 35687300

[6] Regenhardt RW Gonzalez RG He J Lev MH Singhal AB. Symmetric Cta collaterals identify patients with slow-progressing stroke likely to benefit from late thrombectomy. Radiology. (2022) 302:400–7. doi: 10.1148/radiol.2021210455, PMID: 34726532 PMC8792270

[7] Bang OY Saver JL Kim SJ Kim GM Chung CS Ovbiagele B. Collateral flow predicts response to endovascular therapy for acute ischemic stroke. Stroke. (2011) 42:693–9. doi: 10.1161/STROKEAHA.110.595256, PMID: 21233472 PMC3051344

[8] Koneru M Hoseinyazdi M Lakhani DA Greene C Copeland K Wang R. Redefining Ct perfusion-based ischemic core estimates for the ghost core in early time window stroke. J Neuroimaging. (2024) 34:249–56. doi: 10.1111/jon.13180, PMID: 38146065

[9] Pavlina AA Radhakrishnan R Vagal AS. Role of imaging in acute ischemic stroke. Semin Ultrasound CT MR. (2018) 39:412–24. doi: 10.1053/j.sult.2018.01.002, PMID: 30244757

[10] McVerry F Liebeskind DS Muir KW. Systematic review of methods for assessing leptomeningeal collateral flow. AJNR Am J Neuroradiol. (2012) 33:576–82. doi: 10.3174/ajnr.A2794, PMID: 22135128 PMC7966447

[11] Kim SJ Son JP Ryoo S Lee MJ Cha J Kim KH. A novel magnetic resonance imaging approach to collateral flow imaging in ischemic stroke. Ann Neurol. (2014) 76:356–69. doi: 10.1002/ana.24211, PMID: 24985162

[12] de Havenon A Haynor DR Tirschwell DL Majersik JJ Smith G Cohen W. Association of collateral blood vessels detected by arterial spin labeling magnetic resonance imaging with neurological outcome after ischemic stroke. JAMA Neurol. (2017) 74:453–8. doi: 10.1001/jamaneurol.2016.4491, PMID: 28192548 PMC5470363

[13] Alsop DC Detre JA Golay X Gunther M Hendrikse J Hernandez-Garcia L. Recommended implementation of arterial spin-labeled perfusion Mri for clinical applications: a consensus of the Ismrm perfusion study group and the European consortium for Asl in dementia. Magn Reson Med. (2015) 73:102–16. doi: 10.1002/mrm.25197, PMID: 24715426 PMC4190138

[14] De Simone M Fontanella MM Choucha A Schaller K Machi P Lanzino G. Current and future applications of arterial spin labeling Mri in cerebral arteriovenous malformations. Biomedicine. (2024) 12:753. doi: 10.3390/biomedicines12040753, PMID: 38672109 PMC11048131

[15] Qiao PG Han C Zuo ZW Wang YT Pfeuffer J Duan L. Clinical assessment of cerebral hemodynamics in Moyamoya disease via multiple inversion time arterial spin labeling and dynamic susceptibility contrast-magnetic resonance imaging: a comparative study. J Neuroradiol. (2017) 44:273–80. doi: 10.1016/j.neurad.2016.12.006, PMID: 28168990

[16] Ukai R Mikami T Nagahama H Wanibuchi M Akiyama Y Miyata K. Arterial transit artifacts observed by arterial spin labeling in Moyamoya disease. J Stroke Cerebrovasc Dis. (2020) 29:105058. doi: 10.1016/j.jstrokecerebrovasdis.2020.105058, PMID: 32807463

[17] Zaharchuk G Do HM Marks MP Rosenberg J Moseley ME Steinberg GK. Arterial spin-labeling MRI can identify the presence and intensity of collateral perfusion in patients with Moyamoya disease. Stroke. (2011) 42:2485–91. doi: 10.1161/strokeaha.111.616466, PMID: 21799169 PMC3164217

[18] Bolar DS Gagoski B Orbach DB Smith E Adalsteinsson E Rosen BR. Comparison of Cbf measured with combined velocity-selective arterial spin-labeling and pulsed arterial spin-labeling to blood flow patterns assessed by conventional angiography in pediatric Moyamoya. AJNR Am J Neuroradiol. (2019) 40:1842–9. doi: 10.3174/ajnr.A6262, PMID: 31694821 PMC6975103

[19] Lyu J Ma N Liebeskind DS Wang DJ Ma L Xu Y. Arterial spin labeling magnetic resonance imaging estimation of antegrade and collateral flow in unilateral middle cerebral artery stenosis. Stroke. (2016) 47:428–33. doi: 10.1161/STROKEAHA.115.011057, PMID: 26732570 PMC4729602

[20] Lindner T Bolar DS Achten E Barkhof F Bastos-Leite AJ Detre JA. Current state and guidance on arterial spin labeling perfusion Mri in clinical neuroimaging. Magn Reson Med. (2023) 89:2024–47. doi: 10.1002/mrm.29572, PMID: 36695294 PMC10914350

[21] Wu EZ Liu X Dornbos D 3rd Pfeuffer J Qian TY Qiao PG. Comparison of 3D multi-inversion time arterial spin labeling and digital subtraction angiography in the evaluation of cerebral collateral circulation. CNS Neurosci Ther. (2016) 22:1009–11. doi: 10.1111/cns.12603 27650696 PMC6492774

[22] Gunther M Oshio K Feinberg DA. Single-shot 3d imaging techniques improve arterial spin labeling perfusion measurements. Magn Reson Med. (2005) 54:491–8. doi: 10.1002/mrm.20580, PMID: 16032686

[23] Luh WM Wong EC Bandettini PA Hyde JS. Quipss ii with thin-slice Ti1 periodic saturation: a method for improving accuracy of quantitative perfusion imaging using pulsed arterial spin labeling. Magn Reson Med. (1999) 41:1246–54. doi: 10.1002/(sici)1522-2594(199906)41:6<1246::aid-mrm22>3.0.co;2-n, PMID: 10371458

[24] Martin SZ Madai VI von Samson-Himmelstjerna FC Mutke MA Bauer M Herzig CX. 3d Grase pulsed arterial spin labeling at multiple inflow times in patients with long arterial transit times: comparison with dynamic susceptibility-weighted contrast-enhanced Mri at 3 tesla. J Cereb Blood Flow Metab. (2015) 35:392–401. doi: 10.1038/jcbfm.2014.200, PMID: 25407272 PMC4348376

[25] Higashida RT Furlan AJ Roberts H Tomsick T Connors B Barr J. Trial design and reporting standards for intra-arterial cerebral thrombolysis for acute ischemic stroke. Stroke. (2003) 34:e109–37. doi: 10.1161/01.STR.0000082721.62796.09 12869717

[26] Kucinski T Koch C Eckert B Becker V Krömer H Heesen C. Collateral circulation is an independent radiological predictor of outcome after thrombolysis in acute Ischaemic stroke. Neuroradiology. (2003) 45:11–8. doi: 10.1007/s00234-002-0881-0, PMID: 12525948

[27] Lou X Ma X Liebeskind DS Ma N Tian C Lyu J. Collateral perfusion using arterial spin labeling in symptomatic versus asymptomatic middle cerebral artery stenosis. J Cereb Blood Flow Metab. (2019) 39:108–17. doi: 10.1177/0271678X17725212, PMID: 28786338 PMC6311674

[28] Bang OY Saver JL Buck BH Alger JR Starkman S Ovbiagele B. Impact of collateral flow on tissue fate in acute Ischaemic stroke. J Neurol Neurosurg Psychiatry. (2008) 79:625–9. doi: 10.1136/jnnp.2007.132100, PMID: 18077482 PMC2702489

[29] Lou Y Liu Z Ji Y Cheng J Zhao C Li L. Efficacy and safety of very early rehabilitation for acute ischemic stroke: a systematic review and meta-analysis. Front Neurol. (2024) 15:1423517. doi: 10.3389/fneur.2024.1423517, PMID: 39502386 PMC11534803

[30] Shuaib A Butcher K Mohammad AA Saqqur M Liebeskind DS. Collateral blood vessels in acute Ischaemic stroke: a potential therapeutic target. Lancet Neurol. (2011) 10:909–21. doi: 10.1016/S1474-4422(11)70195-8, PMID: 21939900

[31] Clement P Mutsaerts HJ Vaclavu L Ghariq E Pizzini FB Smits M. Variability of physiological brain perfusion in healthy subjects - a systematic review of modifiers. Considerations for multi-center Asl studies. J Cereb Blood Flow Metab. (2018) 38:1418–37. doi: 10.1177/0271678X17702156, PMID: 28393659 PMC6120130

[32] Tan JC Dillon WP Liu S Adler F Smith WS Wintermark M. Systematic comparison of perfusion-Ct and Ct-angiography in acute stroke patients. Ann Neurol. (2007) 61:533–43. doi: 10.1002/ana.21130, PMID: 17431875

[33] Xu Y Guo S Jiang H Han H Sun J Wu X. Collateral status and clinical outcomes after mechanical thrombectomy in patients with anterior circulation occlusion. J Healthc Eng. (2022) 2022:1–7. doi: 10.1155/2022/7796700, PMID: 35126946 PMC8808144

[34] Henderson RD Eliasziw M Fox AJ Rothwell PM Barnett HJ. Angiographically defined collateral circulation and risk of stroke in patients with severe carotid artery stenosis. North American Symptomatic Carotid Endarterectomy Trial (Nascet) Group. Stroke. (2000) 31:128–32. doi: 10.1161/01.str.31.1.128, PMID: 10625727

[35] Reinhard M Roth M Muller T Czosnyka M Timmer J Hetzel A. Cerebral autoregulation in carotid artery occlusive disease assessed from spontaneous blood pressure fluctuations by the correlation coefficient index. Stroke. (2003) 34:2138–44. doi: 10.1161/01.STR.0000087788.65566.AC, PMID: 12920261

[36] Son JP Lee MJ Kim SJ Chung JW Cha J Kim GM. Impact of slow blood filling via collaterals on infarct growth: comparison of mismatch and collateral status. J Stroke. (2017) 19:88–96. doi: 10.5853/jos.2016.00955, PMID: 28030891 PMC5307934

[37] Lin T Lai Z Lv Y Qu J Zuo Z You H. Effective collateral circulation may indicate improved perfusion territory restoration after carotid endarterectomy. Eur Radiol. (2018) 28:727–35. doi: 10.1007/s00330-017-5020-8, PMID: 28894898

[38] Wu B Wang X Guo J Xie S Wong EC Zhang J. Collateral circulation imaging: Mr perfusion territory arterial spin-labeling at 3t. AJNR Am J Neuroradiol. (2008) 29:1855–60. doi: 10.3174/ajnr.A1259, PMID: 18784211 PMC8118955

[39] Chng SM Petersen ET Zimine I Sitoh YY Lim CC Golay X. Territorial arterial spin labeling in the assessment of collateral circulation: comparison with digital subtraction angiography. Stroke. (2008) 39:3248–54. doi: 10.1161/STROKEAHA.108.520593, PMID: 18845805

[40] Hernandez-Garcia L Aramendia-Vidaurreta V Bolar DS Dai W Fernandez-Seara MA Guo J. Recent technical developments in Asl: a review of the state of the art. Magn Reson Med. (2022) 88:2021–42. doi: 10.1002/mrm.29381, PMID: 35983963 PMC9420802

[41] Albers GW Marks MP Kemp S Christensen S Tsai JP Ortega-Gutierrez S. Thrombectomy for stroke at 6 to 16 hours with selection by perfusion imaging. N Engl J Med. (2018) 378:708–18. doi: 10.1056/NEJMoa1713973, PMID: 29364767 PMC6590673

[42] Nogueira RG Jadhav AP Haussen DC Bonafe A Budzik RF Bhuva P. Thrombectomy 6 to 24 hours after stroke with a mismatch between deficit and infarct. N Engl J Med. (2018) 378:11–21. doi: 10.1056/NEJMoa1706442, PMID: 29129157

